# Reply to: A Lethal Progressive Neuroinflammation Disguised as MOGAD Revealing a Final Diagnosis of Griscelli Syndrome: Regarding: MOGAD is the Most Common Cause of Isolated Optic Neuritis in Children

**DOI:** 10.1002/acn3.70471

**Published:** 2026-06-29

**Authors:** Chaitanya Aduru, Akansha Chandrasekar, Kyla Blasingame, Jonathan Rosen, Jesse M. Levine, Karla Salazar, Madhuri Chilakapati, Rod Foroozan, Victoria Adeseye, Jonathan M. Yarimi, Nikita Shukla, Timothy E. Lotze, Kristen S. Fisher, Alexander J. Sandweiss

**Affiliations:** ^1^ Section of Neurology and Developmental Neuroscience, Department of Pediatrics Baylor College of Medicine and Texas Children's Hospital Houston Texas USA; ^2^ Department of Ophthalmology Baylor College of Medicine and Texas Children's Hospital Houston Texas USA

Dear Editor,

1

We thank Dr. Veredice et al. [1] for their interest in our paper “MOGAD is the Most Common Cause of Isolated Optic Neuritis in Children,” and for sharing the devastating and highly instructive case of their 5‐year‐old girl with Griscelli syndrome type 2. We entirely agree with their conclusion that when the clinical picture deviates from classic presentations, clinicians must maintain a high index of suspicion and pursue alternative genetic, structural, or other broader diagnoses.

However, we believe the case presented fundamentally differs from the patient population evaluated in our study, both in its clinical phenotype and its serological diagnostic workup. First, our study specifically focused on *isolated optic neuritis* in the pediatric population. The complete right retinal detachment and partial albinism described in the patient by Dr. Veredice et al. are atypical features that immediately place this patient outside the typical spectrum of isolated optic neuritis or classic MOGAD.

Second, the serological diagnosis of MOGAD in their patient rests on a MOG‐IgG serum titer of 1:10, which in the context of a severe, atypical, and ultimately an alternative neuroinflammatory diagnosis of HLH, is likely to represent a false positive. Additionally, the authors do not state the source of clinical MOG‐IgG testing or if it was conducted via live‐cell or fixed cell assay, which are critically important to the interpretation of results since a fixed cell assay requires a titer of ≥ 1:100 to be considered clear positive [2]. As recently emphasized by Dr. Vilaseca and the Mayo Group in their TRUE‐MOGAD report [3], a minimum titer of 1:20 on a live‐cell assay is required to even apply the diagnostic scoring system, and isolated low‐positive titers are frequently inadequate to confirm true disease without a highly congruent clinical phenotype. By contrast to the patient with Griscelli syndrome type 2, the pediatric patients in our cohort with isolated optic neuritis almost exclusively demonstrated clear positivity (all but one patient in our cohort had a live‐cell assay MOG‐IgG titer of ≥ 1:200) [4].

We appreciate the authors highlighting the ultimate danger of diagnostic anchoring; their case is a sobering reminder that a positive autoantibody test must always be interpreted strictly within the clinical context. Nonetheless, in children presenting with truly *isolated optic neuritis*, our single‐center data support MOGAD as the most common etiology.

## Funding

The authors have nothing to report.

## Conflicts of Interest

The authors declare no conflicts of interest.

## Linked Articles

This Reply links to the following articles: Veredice et al. https://doi.org/10.1002/acn3.70470 and Aduru and Chandrasekar et al. https://doi.org/10.1002/acn3.70422.

## Data Availability

The authors have nothing to report.
